# *In Vitro* Antibacterial and Antifungal Activity of Salicylanilide Benzoates

**DOI:** 10.1100/2012/290628

**Published:** 2012-04-30

**Authors:** Martin Krátký, Jarmila Vinšová, Vladimír Buchta

**Affiliations:** ^1^Department of Inorganic and Organic Chemistry, Faculty of Pharmacy, Charles University, Heyrovského 1203, 500 05 Hradec Králové, Czech Republic; ^2^Department of Clinical Microbiology, Faculty of Medicine and University Hospital, Charles University, Sokolská 581, 500 12 Hradec Králové, Czech Republic; ^3^Department of Biological and Medical Sciences, Faculty of Pharmacy, Charles University, Heyrovského 1203, 500 05 Hradec Králové, Czech Republic

## Abstract

The resistance to antimicrobial agents brings a need of novel antimicrobial agents. We have synthesized and found the *in vitro* antibacterial activity of salicylanilide esters with benzoic acid (2-(phenylcarbamoyl)phenyl benzoates) in micromolar range. They were evaluated *in vitro* for the activity against eight fungal and eight bacterial species. All derivatives showed a significant antibacterial activity against Gram-positive strains with minimum inhibitory concentrations ≥0.98 *μ*mol/L including methicillin-resistant *Staphylococcus aureus* strain. The most active compounds were 5-chloro-2-(3,4-dichlorophenylcarbamoyl)phenyl benzoate and 4-chloro-2-(4-(trifluoromethyl)phenylcarbamoyl)phenyl benzoate. The antifungal activity is significantly lower.

## 1. Introduction

The worldwide epidemic of antibiotic resistance is in danger of ending the “golden age” of antibiotic therapy and therefore is touching all people [1]. Major current problems arise from the spread of nosocomial antibiotic-resistant bacteria such as methicillin-resistant *Staphylococcus aureus* (MRSA), extended-spectrum *β*-lactamases-producing (ESBL) *Escherichia coli* or *Klebsiella *spp., multiresistant *Pseudomonas,* or *Acinetobacter* sp. as well as *Clostridium difficile* [2]. 

The situation in the fungal kingdom is a bit different. Over the past decades there has been a growing number of immunocompromised patients (e.g., patients with AIDS or after transplantations) who can develop opportunistic mycoses caused by expanding spectrum of fungal pathogens, including those with problematic susceptibility to current antifungal drugs. Pathogenic fungi can use different mechanisms of resistance to diverse drugs with unrelated modes of action [3].

The searching for potential antimicrobial agents is still challenging and new groups of compounds are desired [4].

Various salicylanilide (2-hydroxy-*N*-phenylbenzamide) esters have displayed good antibacterial and antifungal activities, especially against Gram-positive strains [5–7]. Recently salicylanilides were described besides an excellent antibacterial acting against both drug-sensitive and methicillin-resistant *S. aureus* inhibition activity towards bacterial transglycosylase, an enzyme necessary for the formation of the cell wall [8].

Benzoic acid alone is known as a nonspecific antimicrobial agent with the wide spectrum of the activities against human pathogenic fungi and bacteria with different minimum inhibitory concentration (MIC) values [9–14]; moreover it was being evaluated as an inhibitor of *β*-carbonic anhydrase, a new molecular target occurring in *C. albicans* and *Cryptococcus neoformans *[15]. The review of the benzoic acid as preservative agent, the mechanisms of action, and resistance were published [16]. 

Based on these facts, we designed and evaluated new salicylanilide benzoates as potential antibacterial and antifungal agents.

## 2. Material and Methods

### 2.1. Chemistry

Salicylanilides were prepared by the procedure described previously [7]. The esters were prepared from salicylanilides by using benzoic acid and *N*,*N *′-dicyclohexylcarbodiimide as dehydrating and condensation agent (e.g., [7]). The general structure is presented in the head of Table 1.

All used chemicals were purchased from commercial sources (Sigma-Aldrich) and they were used without a further purification. Reactions were monitored by thin-layer chromatography plates coated with 0.2 mm silica gel 60 F_254_ (Merck) visualized by UV irradiation (254 nm). All synthesized compounds were characterized. Elemental analysis (C, H, N) was performed on an automatic microanalyser CHNS-O CE instrument (FISONS EA 1110, Italy). Melting points were determined on a Melting Point machine B-540 (Büchi) apparatus using open capillaries and they are uncorrected. Infrared spectra (ATR) were recorded on FT-IR spectrometer Nicolet 6700 FT-IR in the range of 400–4000 cm^−1^. The NMR spectra were recorded on a Varian VNMR S500 (500 MHz for ^1^H and 125 MHz for ^13^C; Varian, Inc., Palo Alto, USA) at ambient temperature using deuterated dimethyl sulfoxide (DMSO-*d6*) solutions of the samples. The chemical shifts *δ* are given in ppm, with respect to tetramethylsilane as an internal standard. The coupling constants (*J*) are reported in Hz.

### 2.2. Biology

#### 2.2.1. Antibacterial Evaluation

The *in vitro* antibacterial activity was assayed against next Gram-positive and Gram-negative strains: *Staphylococcus aureus* CCM 4516/08, methicillin-resistant *Staphylococcus aureus* H 5996/08 (MRSA), *Staphylococcus epidermidis* H 6966/08, *Enterococcus *sp. J 14365/08, *Escherichia coli* CCM4517, *Klebsiella pneumoniae *D 11750/08, ESBL-positive *Klebsiella pneumoniae *J 14368/08, and *Pseudomonas aeruginosa* CCM 1961.

The microdilution broth method modified according to standard M07-A07 [17] in Mueller-Hinton broth (HiMedia Laboratories, India) was adjusted to pH 7.4 (±0.2). The investigated compounds were dissolved in DMSO to the final concentrations ranging from 500 to 0.49 *μ*mol/L. Penicillin G (benzylpenicillin) and benzoic acid were used as comparative standard drugs. Bacterial inoculum in sterile water was prepared to match 0.5 McFarland scale (1.5 × 10^8^ CFU/mL). The minimum inhibitory concentrations (MICs) were assayed as 80% (IC_80_) or higher reduction of growth in comparison to the control. The determination of results was performed visually and spectrophotometrically (at 540 nm). The values of MICs were determined after 24 and 48 h of incubation in the darkness at 35°C (±0.1) in a humid atmosphere. 

#### 2.2.2. Antifungal Evaluation

The inhibitory activity was determined *in vitro* against four yeast strains (*Candida albicans* ATCC 44859, *Candida tropicalis* 156, *Candida krusei* E28, and *Candida glabrata* 20/I) and four moulds (*Trichosporon asahii* 1188, *Aspergillus fumigatus* 231, *Absidia corymbifera* 272, and *Trichophyton mentagrophytes* 445).

The method used was microdilution broth method in the format of the CLSI M27-A3 and M38 A2 guidelines for yeasts and moulds [18, 19] in RPMI 1640 with glutamine (KlinLab, the Czech Republic) buffered to pH 7.0 with 0.165 M of 3-morpholino-propane-1-sulphonic acid (Sigma-Aldrich, Germany). DMSO served as a diluent for all compounds. Fungal inoculum was prepared to give a final concentration of 5 × 10^3^ ± 0.2 CFU/mL. Fluconazole was used as a reference drug. Other conditions were the same as for antibacterial assay; only for *T. mentagrophytes* the final MIC were determined after 72 and 120 h of incubation. MICs were determined twice and in duplicate.

## 3. Results and Discussion

### 3.1. Chemistry

Eighteen new salicylanilide benzoates were synthesized. The yields ranged from 44 to 88%.

**4-Chloro-2-(3-chlorophenylcarbamoyl)phenyl Benzoate (1)**

White solid; yield 82%; mp 146.5–149°C. IR (ATR): 3325 (NH amide; m), 3081, 2932, 2853, 1716 (CO ester; s), 1672 (CO amide; s), 1590, 1525, 1483, 1451, 1424, 1309, 1286, 1267, 1251, 1208, 1181, 1104, 1085, 1067, 1023, 902, 873, 786, 735, 703, 681. ^1^H NMR (500 MHz, DMSO): *δ* 10.69 (1H, bs, NH), 8.07 (2H, d, *J* = 7.5 Hz, H2′′, H6′′), 7.83 (1H, d, *J* = 2.5 Hz, H3), 7.76–7.69 (3H, m, H5, H6, H2′), 7.56 (1H, t, *J* = 7.7 Hz, H4′′), 7.53–7.49 (3H, m, H6′, H3′′, H5′′), 7.31 (1H, t, *J* = 8.1 Hz, H5′), 7.11 (1H, dd, *J* = 1.9 Hz, *J* = 7.9 Hz, H4′). ^13^C NMR (125 MHz, DMSO): *δ* 164.3, 163.0, 147.0, 140.3, 134.4, 133.1, 131.8, 131.2, 130.6, 130.4, 130.0, 129.1, 128.6, 128.0, 125.7, 123.8, 119.4, 118.4. Anal. Calcd. for C_20_H_13_Cl_2_NO_3_ (386.23): C, 62.19; H, 3.39; N, 3.63. Found: C, 61.89; H, 3.50; N, 3.87.

**5-Chloro-2-(3-chlorophenylcarbamoyl)phenyl Benzoate (2)**

White solid; yield 68%; mp 166–168°C. IR (ATR): 3282 (NH amide; m), 3072, 1739 (CO ester; s), 1647 (CO amide; s), 1600, 1589, 1548, 1481, 1450, 1410, 1320, 1255, 1241, 1192, 1075, 1051, 1021, 915, 896, 873, 854, 829, 782, 702, 676, 660. ^1^H NMR (500 MHz, DMSO): *δ* 10.65 (1H, bs, NH), 8.08 (2H, d, *J* = 7.9 Hz, H2′′, H6′′), 7.79 (1H, d, *J* = 8.3 Hz, H3), 7.76–7.67 (3H, m, H4, H6, H2′), 7.58–7.48 (4H, m, H6′, H3′′, H4′′, H5′′) 7.30 (1H, t, *J* = 8.1 Hz, H5′), 7.10 (1H, dd, *J* = 1.8 Hz, *J* = 7.9 Hz, H4′). ^13^C NMR (125 MHz, DMSO): *δ* 164.2, 163.5, 148.9, 140.3, 135.8, 134.4, 133.1, 131.0, 130.6, 130.1, 129.1, 128.6, 128.5, 126.5, 124.0, 123.7, 119.4, 118.4. Anal. Calcd. for C_20_H_13_Cl_2_NO_3_ (386.23): C, 62.19; H, 3.39; N, 3.63. Found: C, 62.34; H, 3.22; N, 3.79.

**4-Chloro-2-(4-chlorophenylcarbamoyl)phenyl Benzoate (3)**

White solid; yield 80%; mp 185–187°C. IR (ATR): 3309 (NH amide; m), 3072, 2928, 2850, 1741 (CO ester; s), 1649 (CO amide; s), 1593, 1543, 1537, 1490, 1451, 1405, 1314, 1257, 1245, 1197, 1099, 1053, 1023, 875, 836, 814, 724, 706, 669. ^1^H NMR (500 MHz, DMSO): *δ* 10.65 (1H, bs, NH), 8.06 (2H, d, *J* = 7.2 Hz, H2′′, H6′′), 7.82 (1H, d, *J* = 2.6 Hz, H3), 7.73–7.68 (2H, m, H5, H6), 7.63 (2H, d, *J* = 8.9 Hz, H2′, H6′), 7.55 (1H, t, *J* = 7.8 Hz, H4′′), 7.50 (2H, t, *J* = 8.7 Hz, H3′′, H5′′), 7.33 (2H, d, *J* = 8.9 Hz, H3′, H5′). ^13^C NMR (125 MHz, DMSO): *δ* 164.3, 162.8, 146.9, 137.8, 134.3, 131.6, 131.3, 130.4, 130.0, 129.1, 128.7, 128.0, 127.7, 127.1, 125.7, 121.5. Anal. Calcd. for C_20_H_13_Cl_2_NO_3_ (386.23): C, 62.19; H, 3.39; N, 3.63. Found: C, 62.00; H, 3.45; N, 3.87.

**5-Chloro-2-(4-chlorophenylcarbamoyl)phenyl Benzoate (4)**

White solid; yield 81%; mp 192–194°C. IR (ATR): 3344 (NH amide; m), 3070, 1746 (CO ester; s), 1650 (CO amide; s), 1592, 1533, 1491, 1453, 1401, 1307, 1259, 1243, 1191, 1176, 1077, 1052, 1021, 915, 893, 827, 762, 703. ^1^H NMR (500 MHz, DMSO): *δ* 10.61 (1H, bs, NH), 8.07 (2H, d, *J* = 7.4 Hz, H2′′, H6′′), 7.78 (1H, d, *J* = 8.3 Hz, H3), 7.73–7.67 (2H, m, H4, H6), 7.63 (2H, d, *J* = 8.8 Hz, H2′, H6′), 7.58–7.53 (3H, m, H3′′, H4′′, H5′′), 7.32 (2H, d, *J* = 8.8 Hz, H3′, H5′). ^13^C NMR (125 MHz, DMSO): *δ* 164.2, 163.3, 148.9, 137.9, 135.7, 134.4, 130.9, 130.1, 129.1, 128.8, 128.7, 128.6, 127.6, 126.5, 123.9, 121.4. Anal. Calcd. for C_20_H_13_Cl_2_NO_3_ (386.23): C, 62.19; H, 3.39; N, 3.63. Found: C, 61.87; H, 3.54; N, 3.90.

**4-Chloro-2-(3,4-dichlorophenylcarbamoyl)phenyl Benzoate (5)**

White solid; yield 52%; mp 169.5–172°C. IR (ATR): 3304 (NH amide; m), 3074, 2928, 2850, 1713 (CO ester; s), 1668 (CO amide; s), 1578, 1516, 1478, 1469, 1449, 1384, 1298, 1275, 1247, 1208, 1101, 1087, 1066, 1026, 889, 866, 704. ^1^H NMR (500 MHz, DMSO): *δ* 10.79 (1H, bs, NH), 8.06 (2H, d, *J* = 7.4 Hz, H2′′, H6′′), 7.92 (1H, s, H2′), 7.84 (1H, d, *J* = 2.6 Hz, H3), 7.74–7.69 (2H, m, H5, H6), 7.58–7.50 (5H, m, H5′, H6′, H3′′, H4′′, H5′′). ^13^C NMR (125 MHz, DMSO): *δ* 164.3, 163.0, 147.0, 138.9, 134.4, 131.9, 131.1, 130.9, 130.4, 130.0, 129.1, 128.6, 128.0, 127.9, 127.0, 125.7, 121.1, 120.0. Anal. Calcd. for C_20_H_12_Cl_3_NO_3_ (420.67): C, 57.10; H, 2.88; N, 3.33. Found: C, 57.40; H, 2.99; N, 3.41.

**5-Chloro-2-(3,4-dichlorophenylcarbamoyl)phenyl Benzoate (6)**

White solid; yield 82%; mp 155–157°C. IR (ATR): 3412 (NH amide; m), 3093, 2930, 2851, 1754 (CO ester; s), 1682 (CO amide; s), 1593, 1527, 1475, 1450, 1400, 1375, 1303, 1244, 1180, 1135, 1042, 1020, 914, 881, 823, 759, 700. ^1^H NMR (500 MHz, DMSO): *δ* 10.75 (1H, bs, NH), 8.07 (2H, d, *J* = 7.9 Hz, H2′′, H6′′), 7.92 (1H, s, H2′), 7.79 (1H, d, *J* = 8.3 Hz, H3), 7.74–7.69 (2H, m, H4, H6), 7.59–7.53 (5H, m, H5′, H6′, H3′′, H4′′, H5′′). ^13^C NMR (125 MHz, DMSO): *δ* 164.2, 163.6, 148.9, 139.0, 136.0, 134.4, 131.1, 130.9, 130.1, 129.1, 128.8, 128.0, 127.1, 126.5, 125.6, 124.0, 121.1, 119.9. Anal. Calcd. for C_20_H_12_Cl_3_NO_3_ (420.67): C, 57.10; H, 2.88; N, 3.33. Found: C, 56.95; H, 2.82; N, 3.59.

**2-(3-Bromophenylcarbamoyl)-4-chlorophenyl Benzoate (7)**

White solid; yield 84%; mp 144–146°C. IR (ATR): 3325 (NH amide; m), 3079, 2930, 2852, 1716 (CO ester; s), 1671 (CO amide; s), 1586, 1520, 1479, 1450, 1419, 1305, 1284, 1266, 1249, 1208, 1103, 1084, 1065, 1023, 893, 872, 783, 733, 702, 684, 672. ^1^H NMR (500 MHz, DMSO): *δ* 10.67 (1H, bs, NH), 8.07 (2H, d, *J* = 7.8 Hz, H2′′, H6′′), 7.89 (1H, s, H2′), 7.83 (1H, d, *J* = 2.5 Hz, H3), 7.73–7.69 (2H, m, H5, H6), 7.58–7.50 (3H, m, H3′′, H4′′, H5′′), 7.45 (1H, d, *J* = 7.4 Hz, H6′), 7.36 (1H, t, *J* = 7.7 Hz, H5′), 7.24 (1H, d, *J* = 5.2 Hz, H4′). ^13^C NMR (125 MHz, DMSO): *δ* 164.3, 163.0, 147.0, 140.4, 134.4, 131.7, 130.9, 130.4, 130.0, 129.1, 128.6, 128.0, 127.1, 126.7, 125.7, 122.3, 121.6, 118.7. Anal. Calcd. for C_20_H_13_BrClNO_3_ (430.68): C, 55.78; H, 3.04; N, 3.25. Found: C, 55.49; H, 3.20; N, 3.48.

**2-(3-Bromophenylcarbamoyl)-5-chlorophenyl Benzoate (8)**

White solid; yield 73%; mp 153.5–156°C. IR (ATR): 3279 (NH amide; m), 1739 (CO ester; s), 1647 (CO amide; s), 1599, 1585, 1541, 1476, 1452, 1407, 1320, 1254, 1241, 1190, 1075, 1050, 1020, 914, 894, 854, 781, 702, 659. ^1^H NMR (500 MHz, DMSO): *δ* 10.63 (1H, bs, NH), 8.08 (2H, d, *J* = 7.5 Hz, H2′′, H6′′), 7.89 (1H, s, H2′), 7.79 (1H, d, *J* = 8.3 Hz, H3), 7.73–7.68 (2H, m, H4, H6), 7.58–7.53 (3H, m, H3′′, H4′′, H5′′), 7.45 (1H, d, *J* = 7.0 Hz, H6′), 7.36 (1H, t, *J* = 7.6 Hz, H5′), 7.23 (1H, d, *J* = 5.2 Hz, H4′). ^13^C NMR (125 MHz, DMSO): *δ* 164.1, 163.5, 148.9, 140.5, 135.8, 134.4, 130.9, 130.1, 129.1, 128.6, 128.0, 127.1, 126.6, 126.5, 124.0, 122.2, 121.6, 118.7. Anal. Calcd. for C_20_H_13_BrClNO_3_ (430.68): C, 55.78; H, 3.04; N, 3.25. Found: C, 55.87; H, 3.31; N, 3.46.

**2-(4-Bromophenylcarbamoyl)-4-chlorophenyl Benzoate (9)**

White solid; yield 79%; mp 199–201°C. IR (ATR): 3308 (NH amide; m), 2932, 1739 (CO ester; s), 1668 (CO amide; s), 1597, 1541, 1487, 1449, 1403, 1314, 1258, 1246, 1197, 1099, 1053, 1023, 875, 833, 813, 706. ^1^H NMR (500 MHz, DMSO): *δ* 10.64 (1H, bs, NH), 8.06 (2H, d, *J* = 7.3 Hz, H2′′, H6′′), 7.82 (1H, d, *J* = 2.5 Hz, H3), 7.73–7.68 (2H, m, H5, H6), 7.59–7.45 (7H, m, H2′, H3′, H5′, H6′, H3′′, H4′′, H5′′). ^13^C NMR (125 MHz, DMSO): *δ* 164.3, 162.8, 147.0, 138.3, 134.4, 131.8, 131.7, 131.3, 130.4, 130.0, 129.1, 129.0, 128.7, 125.6, 121.9, 115.7. Anal. Calcd. for C_20_H_13_BrClNO_3_ (430.68): C, 55.78; H, 3.04; N, 3.25. Found: C, 55.55; H, 3.01; N, 3.51.

**2-(4-Bromophenylcarbamoyl)-5-chlorophenyl Benzoate (10)**

White solid; yield 73%; mp 201–203°C. IR (ATR): 3326 (NH amide; m), 2929, 1745 (CO ester; s), 1651 (CO amide; s), 1601, 1587, 1531, 1487, 1450, 1397, 1260, 1241, 1189, 1176, 1073, 1051, 1020, 914, 893, 823, 810, 762, 703. ^1^H NMR (500 MHz, DMSO): *δ* 10.61 (1H, bs, NH), 8.07 (2H, d, *J* = 7.5 Hz, H2′′, H6′′), 7.78 (1H, d, *J* = 8.3 Hz, H3), 7.73–7.67 (2H, m, H4, H6), 7.59–7.53 (5H, m, H2′, H6′, H3′′, H4′′, H5′′), 7.45 (2H, d, *J* = 8.8 Hz, H3′, H5′). ^13^C NMR (125 MHz, DMSO): *δ* 164.2, 163.3, 148.9, 138.3, 135.7, 134.4, 131.7, 130.9, 130.0, 129.1, 128.7, 128.6, 126.5, 123.9, 121.8, 115.7. Anal. Calcd. for C_20_H_13_BrClNO_3_ (430.68): C, 55.78; H, 3.04; N, 3.25. Found: C, 55.67; H, 2.93; N, 3.29.

**4-Chloro-2-(3-fluorophenylcarbamoyl)phenyl Benzoate (11)**

White solid; yield 61%; mp 143–144.5°C. IR (ATR): 3324 (NH amide; m), 2930, 2852, 1716 (CO ester; s), 1673 (CO amide; s), 1601, 1531, 1485, 1452, 1437, 1316, 1268, 1208, 1176, 1104, 1086, 1067, 1022, 965, 858, 784, 733, 704, 681. ^1^H NMR (500 MHz, DMSO): *δ* 10.71 (1H, bs, NH), 8.07 (2H, d, *J* = 7.9 Hz, H2′′, H6′′), 7.83 (1H, d, *J* = 2.5 Hz, H3), 7.73–7.69 (2H, m, H5, H6), 7.57–7.46 (4H, m, H2′, H3′′, H4′′, H5′′), 7.40–7.29 (2H, m, H5′, H6′), 6.92–6.86 (1H, m, H4′). ^13^C NMR (125 MHz, DMSO): *δ* 164.3, 162.9, 163.1 and 161.2 (*J* = 241.5 Hz), 147.0, 140.6 and 140.5 (*J* = 10.9 Hz), 131.7, 131.2, 130.6 and 130.5 (*J* = 9.5 Hz), 130.4, 130.0, 129.1, 128.6, 128.0, 127.1, 125.7, 115.7 and 115.7 (*J* = 2.5 Hz), 110.6 and 110.5 (*J* = 21.0 Hz), 106.8 and 106.6 (*J* = 26.0 Hz). Anal. Calcd. for C_20_H_13_ClFNO_3_ (369.77): C, 64.96; H, 3.54; N, 3.79. Found: C, 64.80; H, 3.29; N, 3.58.

**5-Chloro-2-(3-fluorophenylcarbamoyl)phenyl Benzoate (12)**

White solid; yield 78%; mp 149–151°C. IR (ATR): 3295 (NH amide; m), 3073, 2931, 1739 (CO ester; s), 1650 (CO amide; s), 1596, 1550, 1489, 1450, 1423, 1324, 1256, 1241, 1196, 1171, 1149, 1075, 1053, 1022, 911, 845, 779, 704, 662. ^1^H NMR (500 MHz, DMSO): *δ* 10.68 (1H, bs, NH), 8.08 (2H, d, *J* = 7.9 Hz, H2′′, H6′′), 7.79 (1H, d, *J* = 8.3 Hz, H3), 7.73–7.68 (2H, m, H4, H6), 7.58–7.51 (4H, m, H2′, H3′′, H4′′, H5′′), 7.39–7.27 (2H, m, H5′, H6′), 6.90–6.85 (1H, m, H4′). ^13^C NMR (125 MHz, DMSO): *δ* 164.2, 163.5, 163.1 and 161.2 (*J* = 241.4 Hz), 148.9, 140.7 and 140.6 (*J* = 11.0 Hz), 135.8, 134.4, 130.9, 130.6 and 130.5 (*J* = 9.4 Hz), 130.1, 129.1, 128.6, 128.5, 126.5, 124.0, 115.7 and 115.6 (*J* = 2.6 Hz), 110.5 and 110.4 (*J* = 21.1 Hz), 106.7 and 106.5 (*J* = 26.1 Hz). Anal. Calcd. for C_20_H_13_ClFNO_3_ (369.77): C, 64.96; H, 3.54; N, 3.79. Found: C, 64.90; H, 3.24; N, 3.99.

**4-Chloro-2-(4-fluorophenylcarbamoyl)phenyl Benzoate (13)**

White solid; yield 88%; mp 149–151°C. IR (ATR): 3318 (NH amide; m), 3076, 2929, 2852, 1715 (CO ester; s), 1665 (CO amide; s), 1622, 1571, 1527, 1505, 1479, 1450, 1406, 1310, 1275, 1250, 1207, 1177, 1151, 1098, 1086, 1064, 1023, 892, 822, 778, 733, 697. ^1^H NMR (500 MHz, DMSO): *δ* 10.56 (1H, bs, NH), 8.07 (2H, d, *J* = 7.2 Hz, H2′′, H6′′), 7.81 (1H, d, *J* = 2.6 Hz, H3), 7.73–7.68 (2H, m, H5, H6), 7.63–7.59 (2H, m, H2′, H6′), 7.55 (1H, t, *J* = 7.8 Hz, H4′′), 7.51–7.48 (2H, m, H3′′, H5′′), 7.14–7.09 (2H, m, H3′, H5′). ^13^C NMR (125 MHz, DMSO): *δ* 164.3, 162.6, 159.5 and 157.5 (*J* = 240.5 Hz), 147.0, 135.2 and 135.2 (*J* = 2.6 Hz), 134.3, 131.5, 130.4, 130.0, 129.1, 128.7, 128.0, 127.1, 125.6, 121.8 and 121.8 (*J* = 7.9 Hz), 115.5 and 115.4 (*J* = 22.2 Hz). Anal. Calcd. for C_20_H_13_ClFNO_3_ (369.77): C, 64.96; H, 3.54; N, 3.79. Found: C, 65.15; H, 3.47; N, 3.70.

**5-Chloro-2-(4-fluorophenylcarbamoyl)phenyl Benzoate (14)**

White solid; yield 84%; mp 142–144°C. IR (ATR): 3295 (NH amide; m), 2930, 2852, 1739 (CO ester; s), 1647 (CO amide; s), 1601, 1547, 1505, 1452, 1411, 1317, 1258, 1245, 1194, 1177, 1155, 1072, 1054, 1023, 893, 835, 826, 706. ^1^H NMR (500 MHz, DMSO): *δ* 10.52 (1H, bs, NH), 8.07 (2H, d, *J* = 7.9 Hz, H2′′, H6′′), 7.78 (1H, d, *J* = 8.3 Hz, H3), 7.73–7.66 (2H, m, H4, H6), 7.63–7.59 (2H, m, H2′, H6′), 7.57–7.53 (3H, m, H3′′, H4′′, H5′′), 7.13–7.08 (2H, m, H3′, H5′). ^13^C NMR (125 MHz, DMSO): *δ* 164.2, 163.1, 159.4 and 157.5 (*J* = 240.5 Hz), 148.9, 135.6, 135.3 and 135.3 (*J* = 2.5 Hz), 134.4, 130.9, 130.0, 129.1, 128.6, 128.0, 126.4, 123.9, 121.8 and 121.7 (*J* = 7.9 Hz), 115.5 and 115.3 (*J* = 22.3 Hz). Anal. Calcd. for C_20_H_13_ClFNO_3_ (369.77): C, 64.96; H, 3.54; N, 3.79. Found: C, 64.74; H, 3.61; N, 3.94.

**4-Chloro-2-(4-(trifluoromethyl)phenylcarbamoyl)phenyl Benzoate (15)**

White solid; yield 44%; mp 179.5–181.5°C. IR (ATR): 3310 (NH amide; m), 1715 (CO ester; s), 1672 (CO amide; s), 1601, 1526, 1476, 1452, 1407, 1317, 1278, 1253, 1207, 1168, 1110, 1086, 1063, 1015, 891, 867, 844, 820, 775, 703. ^1^H NMR (500 MHz, DMSO): *δ* 10.87 (1H, bs, NH), 8.06 (2H, d, *J* = 7.8 Hz, H2′′, H6′′), 7.86 (1H, d, *J* = 2.6 Hz, H3), 7.82 (2H, d, *J* = 8.5 Hz, H2′, H6′), 7.75–7.68 (3H, m, H5, H6), 7.65 (2H, d, *J* = 8.6 Hz, H3′, H5′), 7.57–7.51 (3H, m, H3′′, H4′′, H5′′). ^13^C NMR (125 MHz, DMSO): *δ* 164.3, 163.2, 147.0, 142.5, 134.4, 131.9, 131.1, 130.4, 130.0, 129.2, 129.1, 128.6, 126.8 (q, *J* = 30.5 Hz), 126.2 (d, *J* = 3.7 Hz), 125.7, 124.4 (q, *J* = 271.4 Hz), 119.9. Anal. Calcd. for C_21_H_13_ClF_3_NO_3_ (419.78): C, 60.08; H, 3.12; N, 3.34. Found: C, 60.31; H, 2.87; N, 3.09.

**5-Chloro-2-(4-(trifluoromethyl)phenylcarbamoyl)phenyl Benzoate (16)**

White solid; yield 80%; mp 177.5–179.5°C. IR (ATR): 3325 (NH amide; m), 2927, 1746 (CO ester; s), 1654 (CO amide; s), 1598, 1534, 1481, 1450, 1408, 1323, 1243, 1192, 1160, 1104, 1067, 1048, 1020, 916, 894, 845, 825, 763, 702. ^1^H NMR (500 MHz, DMSO): *δ* 10.84 (1H, bs, NH), 8.07 (2H, d, *J* = 7.2 Hz, H2′′, H6′′), 7.83–7.79 (3H, m, H3, H2′, H6′), 7.71–7.67 (2H, m, H4, H6), 7.64 (2H, d, *J* = 8.1 Hz, H3′, H5′), 7.60–7.54 (3H, m, H3′′, H4′′, H5′′). ^13^C NMR (125 MHz, DMSO): *δ* 164.2, 163.7, 149.0, 142.5, 135.9, 134.4, 131.0, 130.0, 129.1, 128.5, 128.5, 126.5, 126.2 (q, *J* = 3.8 Hz), 124.6 (q, *J* = 285.3 Hz), 124.0, 123.9 (q, *J* = 32.0 Hz), 119.8. Anal. Calcd. for C_21_H_13_ClF_3_NO_3_ (419.78): C, 60.08; H, 3.12; N, 3.34. Found: C, 60.22; H, 3.21; N, 3.43.

**4-Chloro-2-(3-(trifluoromethyl)phenylcarbamoyl)phenyl Benzoate (17)**

White solid; yield 72%; mp 159.5–162°C. IR (ATR): 3314 (NH amide; m), 2931, 2853, 1714 (CO ester; s), 1670 (CO amide; s), 1597, 1540, 1443, 1326, 1274, 1206, 1166, 1119, 1097, 1084, 1064, 1023, 906, 800, 735, 695. ^1^H NMR (500 MHz, DMSO): *δ* 10.83 (1H, bs, NH), 8.07 (2H, d, *J* = 7.2 Hz, H2′′, H6′′), 8.00 (1H, s, H2′), 7.87 (1H, d, *J* = 2.6 Hz, H3), 7.74–7.68 (2H, m, H5, H6), 7.56–7.50 (4H, m, H6′, H3′′, H4′′, H5′′), 7.46–7.35 (2H, m, H4′, H5′). ^13^C NMR (125 MHz, DMSO): *δ* 164.3, 163.1, 147.0, 139.6, 134.4, 131.8, 131.1, 130.4, 130.2, 130.0, 129.6 (q, *J* = 31.7 Hz), 129.1, 128.6, 128.0, 125.7, 124.2 (q, *J* = 272.6 Hz), 123.6, 120.4 (q, *J* = 3.6 Hz), 116.1 (q, *J* = 3.9 Hz). Anal. Calcd. for C_21_H_13_ClF_3_NO_3_ (419.78): C, 60.08; H, 3.12; N, 3.34. Found: C, 60.19; H, 3.35; N, 2.99.

**4-Bromo-2-(4-(trifluoromethyl)phenylcarbamoyl)phenyl Benzoate (18)**

White solid; yield 79%; mp 177.5–180°C. IR (ATR): 3309 (NH amide; m), 1714 (CO ester; s), 1672 (CO amide; s), 1600, 1526, 1474, 1451, 1406, 1317, 1280, 1252, 1208, 1169, 1111, 1086, 1064, 1041, 862, 843, 818, 720, 703. ^1^H NMR (500 MHz, DMSO): *δ* 10.87 (1H, bs, NH), 8.06 (2H, d, *J* = 7.2 Hz, H2′′, H6′′), 7.97 (1H, d, *J* = 2.4 Hz, H3), 7.85 (1H, dd, *J* = 2.5 Hz, *J* = 8.6 Hz, H5), 7.82 (2H, d, *J* = 8.5 Hz, H2′, H6′), 7.70 (1H, t, *J* = 7.5 Hz, H4′′), 7.65 (2H, d, *J* = 8.2 Hz, H3′, H5′), 7.54 (2H, t, *J* = 7.9 Hz, H3′′, H5′′), 7.45 (1H, d, *J* = 8.6 Hz, H6). ^13^C NMR (125 MHz, DMSO): *δ* 164.2, 163.1, 147.5, 142.5, 134.8, 134.4, 132.0, 131.4, 130.0, 129.1, 128.6, 126.7 (q, *J* = 37.3 Hz), 126.2 (d, *J* = 3.7 Hz), 126.0, 124.4 (q, *J* = 271.5 Hz), 119.9, 118.5. Anal. Calcd. for C_21_H_13_BrF_3_NO_3_ (464.23): C, 54.33; H, 2.82; N, 3.02. Found: C, 54.16; H, 3.03; N, 3.35.

### 3.2. Biology

#### 3.2.1. Antibacterial Evaluation

Salicylanilide benzoates were assayed for their *in vitro* antibacterial activity towards eight strains. Benzoic acid expressed no activity up to 500 *μ*mol/L. Table 1 summarizes the results with respect to structure of the esters.

Almost all benzoates exhibited a very good activity against both strains of *S. aureus* with MIC from 0.98 to 31.25 *μ*mol/L (**10** being an exception). Importantly, MIC for drug-sensitive and MRSA strain did not differ practically, which indicates none cross resistance. Also *S. epidermidis* was affected by the majority of evaluated compounds (without **3**) at only slightly higher concentrations from 1.95 *μ*mol/L. *Enterococcus* was the most insensitive Gram-positive strain, although it was inhibited by four esters (**5**, **6**, **15**, **18**) at 1.95 *μ*mol/L; six esters (**2**, **3**, **4**, **10**, **14**, **16**) were inactive at 125 *μ*mol/L. Molecules **6** and **15** were found having the highest *in vitro* inhibitory potency. MIC values against three *Staphylococci* support the hypothesis that these derivatives act as bactericidal agents. 

Some salicylanilide benzoates MIC values are comparable to benzylpenicillin towards *S. aureus*, but almost all esters are favorable against MRSA and *S*. *epidermidis*. The situation for *Enterococcus* is quite more complex; some benzoates exhibited better MIC value (**5**, **6**, **15**, **18**), some comparable after 24 h (**7**, **8**, **12**, **17**) and other worse than benzylpenicillin. 

When concentrated on the structure-activity relationships, the position of the substituents on the salicylic ring is ambiguous—in some cases are superior 4-chloro derivatives (e.g., **1** versus **2**, **9** versus **10**, **15** versus **16**), in other 5-chloro ones (**5** versus **6** or **7** versus **8**). For the substitution of the aniline ring, 3,4-dichloro (**5**, **6**) and CF_3_- (**15**–**18**) moieties improved the antibacterial activity the most significantly. In general, it seems that 3-substituted anilines produced a higher antibacterial activity than 4-substituted anilines. 

Gram-negative species (*E. coli*, two strains of *Klebsiella pneumoniae*, *P. aeruginosa*) were almost completely resistant to benzoates up to 125 *μ*mol/L at the testing conditions with one notable exception; *P. aeruginosa* was inhibited by **1** at low concentration of 3.9/7.81 *μ*mol/L. 

#### 3.2.2. Antifungal Evaluation

Synthesized esters were tested for their *in vitro* activity against eight human pathogenic fungi. MIC values are presented in Table 2. Benzoic acid alone was completely inactive at the concentration of 500 *μ*mol/L at both neutral and slightly acidic (pH ~ 5) environment. 

Unforeseen, in contrast to the antibacterial activity, salicylanilide benzoates expressed only mild antifungal potency. From eight strains, *C. tropicalis*, *C. glabrata*, and *A. fumigatus* showed a complete insensitivity to all tested derivatives at the value of 125 *μ*mol/L. When concentrated on the esters, benzoates **2** and **11** did not affect the growth of any fungal species at the concentration of 500 *μ*mol/L and lower, derivatives **12**, **14,** and **17** up to 250 *μ*mol/L and **3**, **4**, **5**, **7**, **10**, **15**, **16** expressed activity levels >125 *μ*mol/L. Only six derivatives (**1**, **6**, **8**, **9**, **13**, **18**) displayed certain *in vitro* efficacy. The most active compound was assayed trichlorinated ester **6** (MIC ≥ 3.9 *μ*mol/L), which surpassed standard fluconazole against *C. krusei*. In general, *Candida* is more resistant to salicylanilide esters than moulds and moreover, it seems that the mechanism of the action is only fungistatic. On the other side, the growth of *T. mentagrophytes* was affected by the highest number of the derivatives; based on the MIC values, activity against filamentous fungi is probably fungicidal. 

Because pH effect on the efficiency of benzoic acid derivatives (generally of weak organic acids) was described (e.g., [16]), we measured MIC values of the esters **6** and **15** not only at approximately neutral environment, but additionally at slightly acidic pH (~5; without buffering of the testing medium). Unfortunately, the change of pH did not result in the improvement of activity, even it led to a bit worse values. 

Although we expected that the introduction of benzoyl fragment into salicylanilide molecules resulted in the significantly increased antifungal potency, this modification failed in this point. 

## 4. Conclusion

In sum, we have designed and synthesized new esters of halogenated salicylanilides with benzoic acid. This series of compounds was evaluated to be a new group with promising *in vitro* antibacterial (against Gram-positive strains) activity. Unfortunately these derivatives disappointed expectation about them as potential antifungal agents. 

## Figures and Tables

**Table 1:** Antibacterial activity of benzoates **1-18**.

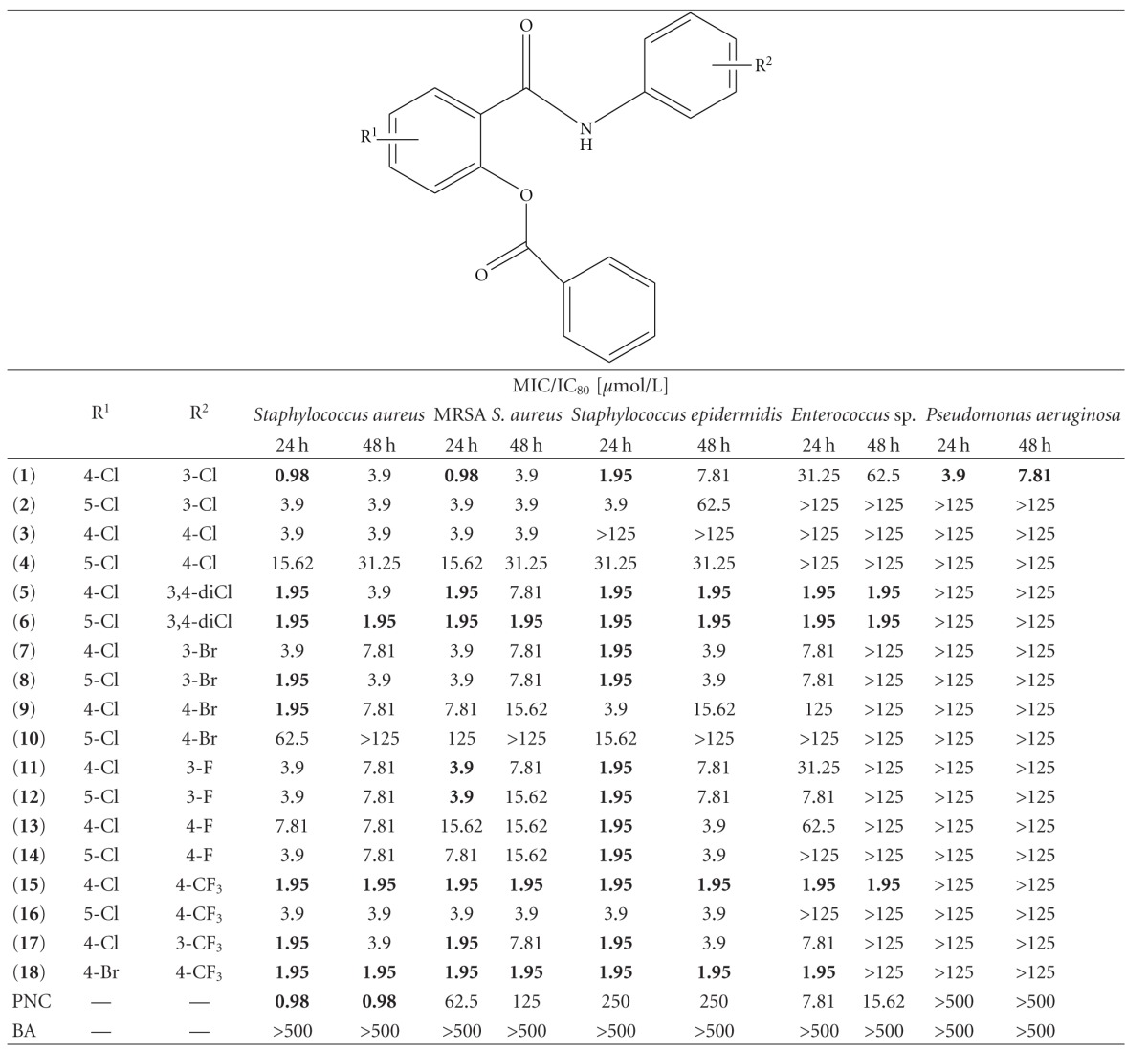

PNC: penicillin G; BA: benzoic acid. The lowest MIC value(s) for each strain are bolded.

**Table 2:** *In vitro* antifungal activity of salicylanilide benzoates.

	MIC/IC_80_ [*μ*mol/L]									
	*Candida albicans*		*Candida krusei*		*Trichosporon asahii*		*Absidia corymbifera*		*Trichophyton mentagrophytes*	
	24 h	48 h	24 h	48 h	24 h	48 h	24 h	48 h	72 h	120 h

**(1)**	250	>500	125	>500	>500	>500	500	>500	250	>500
**(6)**	**125**	>125	**15.62**	>125	**3.9**	**15.62**	125	125	**31.25**	**31.25**
**(8)**	>250	>250	>250	>250	>250	>250	250	>250	>250	>250
**(9)**	>125	>125	>125	>125	>125	>125	125	125	62.5	62.5
**(13)**	>125	>125	>125	>125	>125	>125	**62.5**	>125	125	125
**(18) **	>125	>125	>125	>125	>125	>125	>125	>125	62.5	125
BA	>500	>500	>500	>500	>500	>500	>500	>500	>500	>500
FLU	1.00	2.00	>50.0	>50.0	4.00	9.00	>50.0	>50.0	17.0	26.0

FLU: fluconazole; BA: benzoic acid. The best MIC value for each strain is bolded.
